# Body size and weight change over adulthood and risk of breast cancer by menopausal and hormone receptor status: a pooled analysis of 20 prospective cohort studies

**DOI:** 10.1007/s10654-020-00688-3

**Published:** 2020-10-30

**Authors:** Piet A. van den Brandt, Regina G. Ziegler, Molin Wang, Tao Hou, Ruifeng Li, Hans-Olov Adami, Claudia Agnoli, Leslie Bernstein, Julie E. Buring, Yu Chen, Avonne E. Connor, A. Heather Eliassen, Jeanine M. Genkinger, Gretchen Gierach, Graham G. Giles, Gary G. Goodman, Niclas Håkansson, Vittorio Krogh, Loic Le Marchand, I-Min Lee, Linda M. Liao, M. Elena Martinez, Anthony B. Miller, Roger L. Milne, Marian L. Neuhouser, Alpa V. Patel, Anna Prizment, Kim Robien, Thomas E. Rohan, Norie Sawada, Leo J. Schouten, Rashmi Sinha, Rachael Z. Stolzenberg-Solomon, Lauren R. Teras, Shoichiro Tsugane, Kala Visvanathan, Elisabete Weiderpass, Kami K. White, Walter C. Willett, Alicja Wolk, Anne Zeleniuch-Jacquotte, Stephanie A. Smith-Warner

**Affiliations:** 1 Department of Epidemiology, GROW – School for Oncology and Developmental Biology, Maastricht University, PO Box 616, 6200 MD Maastricht, The Netherlands; 2 Department of Epidemiology, Care and Public Health Institute (CAPHRI), Maastricht University, PO Box 616, 6200 MD Maastricht, The Netherlands; 3 Division of Cancer Epidemiology and Genetics, National Cancer Institute, National Institutes of Health, Bethesda, MD USA; 4 Department of Epidemiology, Harvard T.H. Chan School of Public Health, Boston, MA USA; 5 Department of Biostatistics, Harvard T.H. Chan School of Public Health, Boston, MA USA; 6 Channing Division of Network Medicine, Department of Medicine, Harvard Medical School, Boston, MA USA; 7 Department of Nutrition, Harvard T.H Chan School of Public Health, Boston, MA USA; 8 Department of Medical Epidemiology and Biostatistics, Karolinska Institutet, Stockholm, Sweden; 9 Clinical Effectiveness Group, Institute of Health, University of Oslo, Oslo, Norway; 10 Epidemiology and Prevention Unit, Department of Research, Fondazione Istituto Nazionale Tumori, 20133 Milan, Italy; 11 Department of Population Sciences, Beckman Research Institute, City of Hope, Duarte, CA USA; 12 Division of Preventive Medicine, Brigham and Women’s Hospital, Harvard Medical School, Boston, MA USA; 13 Division of Epidemiology, Department of Population Health and Department of Environmental Medicine, New York University School of Medicine, New York, USA; 14 Department of Epidemiology, Johns Hopkins Bloomberg School of Public Health, Baltimore, MD USA; 15 Department of Epidemiology, Columbia University Mailman School of Public Health, New York, NY USA; 16 Herbert Irving Comprehensive Cancer Center, Columbia University Irving Medical Center, New York, NY USA; 17 Cancer Epidemiology Division, Cancer Council Victoria, Melbourne, VIC Australia; 18 Centre for Epidemiology and Biostatistics, Melbourne School of Population and Global Health, The University of Melbourne, Melbourne, VIC Australia; 19 Precision Medicine, School of Clinical Sciences at Monash Health, Monash University, Clayton, VIC Australia; 20 Division of Public Health Sciences, Fred Hutchinson Cancer Research Center, Seattle, WA USA; 21 Unit of Cardiovascular and Nutritional Epidemiology, Institute of Environmental Medicine, Karolinska Institutet, Stockholm, Sweden; 22 Cancer Epidemiology Program, University of Hawaii Cancer Center, Honolulu, HI USA; 23 Department of Family Medicine and Public Health School of Medicine, University of California San Diego, La Jolla, CA USA; 24 Moores Cancer Center, University of California San Diego, La Jolla, CA USA; 25 Dalla Lana School of Public Health, University of Toronto, Toronto, ON Canada; 26 Epidemiology Research Program, American Cancer Society, Atlanta, GA USA; 27 Division of Hematology, Oncology and Transplantation, University of Minnesota Medical School, Minneapolis, MN USA; 28 Division of Epidemiology and Community Health, School of Public Health, University of Minnesota, Minneapolis, MN USA; 29 Department of Exercise and Nutrition Sciences, Milken Institute School of Public Health, George Washington University, Washington, DC USA; 30 Department of Epidemiology and Population Health, Albert Einstein College of Medicine, Bronx, USA; 31 Epidemiology and Prevention Group, Research Center for Cancer Prevention and Screening, National Cancer Center, Tokyo, Japan; 32 International Agency for Research on Cancer, World Health Organization, Lyon, France; 33 Department of Surgical Sciences, Uppsala University, Uppsala, Sweden

**Keywords:** Breast neoplasms, Body height, Body weight, Weight change, Estrogen receptor, Cohort studies

## Abstract

Associations between anthropometric factors and breast cancer (BC) risk have varied inconsistently by estrogen and/or progesterone receptor (ER/PR) status. Associations between prediagnostic anthropometric factors and risk of premenopausal and postmenopausal BC overall and ER/PR status subtypes were investigated in a pooled analysis of 20 prospective cohorts, including 36,297 BC cases among 1,061,915 women, using multivariable Cox regression analyses, controlling for reproductive factors, diet and other risk factors. We estimated dose–response relationships and tested for nonlinear associations using restricted cubic splines. Height showed positive, linear associations for premenopausal and postmenopausal BC risk (6–7% RR increase per 5 cm increment), with stronger associations for receptor-positive subtypes. Body mass index (BMI) at cohort baseline was strongly inversely associated with premenopausal BC risk, and strongly positively—and nonlinearly—associated with postmenopausal BC (especially among women who never used hormone replacement therapy). This was primarily observed for receptor-positive subtypes. Early adult BMI (at 18–20 years) showed inverse, linear associations for premenopausal and postmenopausal BC risk (21% and 11% RR decrease per 5 kg/m^2^, respectively) with stronger associations for receptor-negative subtypes. Adult weight gain since 18–20 years was positively associated with postmenopausal BC risk, stronger for receptor-positive subtypes, and among women who were leaner in early adulthood. Women heavier in early adulthood generally had reduced premenopausal BC risk, independent of later weight gain. Positive associations between height, baseline (adult) BMI, adult weight gain and postmenopausal BC risk were substantially stronger for hormone receptor-positive versus negative subtypes. Premenopausal BC risk was positively associated with height, but inversely with baseline BMI and weight gain (mostly in receptor-positive subtypes). Inverse associations with early adult BMI seemed stronger in receptor-negative subtypes of premenopausal and postmenopausal BC.

**Electronic supplementary material:**

The online version of this article (10.1007/s10654-020-00688-3) contains supplementary material, which is available to authorized users.

## Introduction

Body height and weight have been consistently reported to be associated with breast cancer (BC) risk [1, 2]. Height generally shows positive associations with both premenopausal and postmenopausal BC [1, 3–5]. Adult relative weight, as measured with body mass index (BMI) and weight gain since adolescence (18–20 years) are inversely associated with premenopausal BC risk, but positively associated with postmenopausal BC [1, 2, 6–11]. Early adult weight has been inversely, although inconsistently, related to both premenopausal and postmenopausal BC risk [11, 12].

Increasingly BC is recognized as a heterogeneous disease with distinct risk factors for tumor subtypes. Investigating how the anthropometry relationships with BC vary by steroid hormone receptor status may provide insight into BC etiology. For weight, the influence on postmenopausal BC risk seems related to hormonal pathways (steroids/estrogens) [13–17]. For postmenopausal BC, adult BMI may have a larger impact on risk of estrogen receptor positive (ER+) BC than ER− tumors, because ER− tumors are less dependent on estrogen levels [14, 18]. However, studies of the associations between BMI and BC according to estrogen and/or progesterone receptor (PR) status [8, 17, 19, 20] have not been entirely consistent [19, 20]. Associations with ER/PR subtypes have been investigated less extensively for early adult BMI, adult weight gain and height.

Meta-analyses [11, 20] of the associations between height, body weight, and weight change with BC risk indicate significant heterogeneity in the study-specific results and publication bias which hinders causal interpretation. Potential sources of heterogeneity include differences in how the exposures are (measured and) modeled and which covariates are included in the models. Meta-analyses of these exposures with BC subtypes defined by hormone receptor status are also limited by the relatively few studies that have reported on these associations. Furthermore, meta-analyses often assume linear associations (e.g., [2, 11]). These limitations of meta-analyses can be directly addressed in pooled analyses of individual data from multiple cohorts, because studies that have not previously published on the association can be included in the analysis thereby limiting publication bias. Furthermore, there is enhanced standardization of the exposures, outcomes, covariates, and modeling approach used. We therefore investigated detailed dose–response relationships between anthropometry and risk of BC by ER status, PR status, and joint ER/PR status in a large pooled analysis of the participant-level data from 20 prospective cohorts, including over 1 million women with more than 36,000 incident BC cases.

## Subjects and methods

### Study population

The Pooling Project of Prospective Studies of Diet and Cancer (DCPP) has been described previously [21]. For these analyses, we included 20 prospective cohorts [9, 10, 22–39] which met the following inclusion criteria: ascertainment of at least 25 incident cases of invasive ER− BC, prospective assessment of anthropometry and long-term dietary intake, and evaluation of the validity of the dietary assessment method or a closely related method. Each participating cohort, participating registries (as required), and the DCPP received approval from their respective institutional review boards.

### Ascertainment of BC cases

Incident invasive BC cases were identified in the cohort studies through follow-up questionnaires and confirmed with subsequent medical record review [9, 32, 36] or linkage with cancer registries [10, 22–24, 26, 27, 33, 35, 37, 40–42], both approaches [28, 31, 34, 39, 43], as well as through mortality registries [23, 26, 28, 32, 39, 43]. We used the steroid hormone receptor status data provided by each cohort to define BC subtypes. We classified cases with borderline ER/PR status as positive for that receptor [44]. Approximately 28% of cases were missing ER/PR status, which were mostly from diagnoses prior to 2000.

### Anthropometry and BC risk factors

Prediagnostic anthropometry, menstrual and reproductive factors, medical history, family history of BC, dietary intake and physical activity were assessed by the cohorts at baseline with self-administered questionnaires. However, CARET, MCCS and ORDET measured height and weight. Weight at early adulthood (ages 18–20 years) was available for 15 cohorts (Table 1, including cohort abbreviations). BMI (kg/m^2^) was used as an indicator of adiposity in mid to later adulthood (‘baseline BMI’) and at ages 18–20 years (‘early adult BMI’). Adult weight change (kg) was defined as the difference between cohort baseline weight and early adult weight.

**Table 1:** Characteristics of the cohort studies included in the pooled analyses of anthropometry and breast cancer risk by estrogen receptor (ER) and progesterone receptor (PR) status, Pooling Project of Prospective Studies of Diet and Cancer

Study (country)	Acronym	Baseline cohort size*	Age range at baseline (years)	Years of follow-up	Breast cancer cases			Height (m)		Baseline BMI (kg/m^2^)		Early adult BMI (kg/m^2^) (at ages 18–20 years)	
					Total	Premenopausal	Postmenopausal	Median	(10–90%)	Median	(10–90%)	Median	(10–90%)
Beta-Carotene and Retinol Efficacy Trial (USA)	CARET	5939	50–69	1985–2005	363	0	363	1.63	1.55–1.70	25.7	20.8–33.8		
Breast Cancer Detection Demonstration Project Follow-up Study (USA)	BCDDP	38,846	40–93	1987–1999	1219	14	1115	1.63	1.55–1.70	24.2	20.3–31.0		
California Teachers Study (USA)	CTS	96,416	22–104	1995–2003	2579	349	1791	1.65	1.57–1.73	23.6	19.8–31.5	20.8	18.3–25.0
Canadian National Breast Screening Study (Canada)	CNBSS	44,671	40–59	1980–2000	1228	332	419	1.63	1.55–1.70	23.8	20.4–30.1	20.8	18.3–24.3
Cancer Prevention Study II Nutrition Cohort (USA)	CPSII	72,999	50–74	1992–2003	2952	14	2767	1.63	1.57–1.73	24.8	20.5–31.8	20.3	17.8–24.0
CLUE II: Campaign Against Cancer and Heart Disease (USA)	CLUE II	8263	18–93	1989–2007	287	35	204	1.63	1.55–1.70	24.8	20.2–32.5	20.8	18.1–25.7
Iowa Women’s Health Study (USA)	IWHS	34,536	55–69	1986–2004	1848	0	1848	1.63	1.55–1.70	25.2	20.8–32.5	20.5	17.8–24.5
Japan Public Health Center-Based Prospective Study 1 (Japan)	JPHC1	21,468	40–59	1990–2004	288	69	145	1.52	1.45–1.58	23.3	19.9–27.6		
Melbourne Collaborative Cohort Study (Australia)	MCCS	22,429	31–75	1990–2006	799	108	470	1.60	1.52–1.69	25.7	21.3–32.9	21.1	18.2–25.0
Multiethnic Cohort (USA)	MEC	90,394	45–75	1993–2004	3261	129	2774	1.60	1.52–1.70	25.0	20.0–33.2	20.3†	17.7–24.2
Netherlands Cohort Study (Netherlands)	NLCS	62,573	55–69	1986–1999	1959	0	1959	1.65	1.57–1.74	24.7	21.2–29.7	21.2	18.1–24.5
New York University Women’s Health Study (USA)	NYUWHS	13,147	34–65	1985–2003	912	193	479	1.63	1.55–1.70	24.0	20.3–30.9		
NIH-AARP Diet and Health Study (USA)	NIH-AARP	192,423	50–71	1995–2003	5768	6	5378	1.63	1.55–1.73	25.8	21.0–34.0	20.4	17.9–24.2
Nurses’ Health Study (a) (USA)	NHSa	88,024	34–67	1980–1986	1113	438	442	1.63	1.57–1.73	23.3	20.0–30.2	20.9	18.3–25.0
Nurses’ Health Study (b) (USA) ‡	NHSb	66,814	40–67	1986–2006	4358	284	2617	1.63	1.57–1.73	24.2	20.4–31.6	20.9	18.3–25.0
Nurses’ Health Study II (USA)	NHS II	90,922	26–46	1991–2003	1289	1112	38	1.65	1.57–1.73	23.2	19.7–31.6	20.6	18.0–25.1
Hormones and Diet in the Etiology of Breast Cancer (Italy)	ORDET	8958	35–69	1987–2002	280	82	128	1.57	1.50–1.65	24.6	20.5–30.9	20.9	18.0–25.0
Prostate, Lung, Colorectal, and Ovarian Cancer Screening Trial (USA)	PLCO	27,992	55–74	1993–2007	1082	0	1082	1.63	1.55–1.70	26.2	21.4–34.3	21.0	18.4–24.5
Swedish Mammography Cohort (Sweden)	SMC	58,668	40–76	1987–2005	2508	172	869	1.64	1.57–1.72	24.1	20.5–29.8		
Women’s Health Study (USA)	WHS	37,568	45–89	1993–2004	1161	74	716	1.65	1.57–1.73	24.9	20.8–32.6		
Women’s Lifestyle and Health Study (Sweden)	WLHS	45,679	30–49	1991–2006	1043	468	52	1.66	1.59–1.73	22.8	19.8–28.0	20.2	17.7–23.7
Total		1,061,915			36,297	3879	25,656						

*Cohort size after applying study-specific exclusion criteria and then excluding women with previous cancer diagnosis (other than nonmelanoma skin cancer); the Netherlands Cohort Study was analyzed as a case-cohort study and the above exclusions were not applied to its baseline cohort size

^†^BMI at age 21 years

^‡^Nurses’ Health Study (b) is not included as part of total cohort size because they are included in Nurses’ Health Study (a). See Methods for further explanation

### Statistical analyses

Participants were excluded from these analyses if at baseline they reported a personal history of cancer except for non-melanoma skin cancer at baseline, if they had missing data on height or baseline weight or if their reported energy intake was greater than three standard deviations from the study-specific log_e_-transformed mean energy intake of the baseline population (an exclusion applied to all cohorts in the DCPP [21]).

Study-specific relative risks (RRs) and 95% confidence intervals (CIs) for total, ER−, ER+, PR− and PR+ BC, and joint ER/PR subtypes, were estimated separately in each study by fitting Cox proportional hazards models [45]. No estimates were calculated for the ER− PR+ subtype due to insufficient case numbers. Cohorts were excluded from analyses of a receptor subtype if there were < 25 cases with that subtype. We calculated person-years of follow-up from the date of questionnaire completion (baseline) to the date of diagnosis of first incident invasive BC, death, loss to follow up, or end of follow-up, whichever came first. We modeled age at baseline (in years) and year of questionnaire completion as stratification variables to adjust simultaneously for age, calendar time, and time since entry into the study [21]. We analyzed the NLCS as a case-cohort study because questionnaires were processed for only the cases and a random sample of the total cohort [46]. As before [47], we analyzed the NHS as two different cohorts (1980–1986, NHS[a]; 1986–2006, NHS[b]) to utilize the comprehensive dietary assessment administered in 1986.

Anthropometric variables (height, baseline BMI, early adult BMI, adult weight change) were entered into models as categorical or, if not statistically significant non-linear, as continuous variables. Baseline BMI was included in the height models; height was included in the baseline BMI, early adult BMI, and adult weight change models. To avoid collider bias [48], baseline BMI was not included in the early adult BMI and adult weight change models. We evaluated whether the observed association between each anthropometric variable and BC risk was modified by menopausal status at diagnosis as estimated using a previously described algorithm based on age at diagnosis [49]. Because postmenopausal hormone replacement therapy (HRT) modifies the relationship between adiposity and BC [6], we also performed analyses for postmenopausal BC according to HRT use (never vs. ever, at baseline). We used meta-regression to test for interactions by menopausal status and HRT use.

In all analyses presented, associations were essentially similar in age-adjusted and multivariable models; therefore, we only present the multivariable results. Multivariable RRs were adjusted for ethnicity, age at menarche, parity, age at first birth, HRT use (for analyses not stratified on this variable), oral contraceptive use, history of benign breast disease, family history of BC, smoking status, education, physical activity, alcohol and energy intake (covariate categories are presented in table footnotes and figure legends). For each covariate, we created a missing indicator variable since the proportion of missing data in these cohorts is generally low [21]. We either adjusted for the abovementioned covariates directly in the model or we adjusted for confounders using the propensity score method [50, 51] when the number of cases of the outcome evaluated within a study was < 200.

We pooled the study-specific RRs weighted by the inverse of their variances using a random effects model [52, 53] and tested for between-studies heterogeneity using the Q statistic [53]. To test for trend across categories of anthropometric variables, we assigned each category its median value and modeled that variable as a continuous term. We used a contrast test [54] to examine whether the associations differed significantly for BC subtypes defined by hormone receptor status (ER− vs. ER+; PR− vs. PR+; ER− PR− vs. ER+ PR− vs. ER+ PR +). All statistical tests were two-sided with a *p* value of 0.05 as significance level, and were conducted using SAS (SAS Institute, Inc., Version 9.4, Cary, NC).

We estimated dose–response relationships of anthropometric variables with BC (subtypes) and tested for non-linear associations using restricted cubic splines [55] with four knots (placed at the 5th, 35th, 65th, and 95^th^ percentiles). In these analyses, we combined all studies into one aggregated dataset, stratified by study, age at baseline, and year of baseline questionnaire, and adjusted for the abovementioned confounding variables. We excluded the top and bottom 1% of the data from the analysis, truncated the spline presentations at 1% and 99% of the distribution, and used approximately the median value for the referent category in the categorical analysis as the spline referent. To assess non-linearity, we used a likelihood ratio test to compare the model including the linear and cubic spline terms with the model including only the linear term for the anthropometric variable of interest. If the assumption of linearity held for the association between the anthropometric variable and BC risk, we further analyzed that anthropometric variable as a continuous variable.

## Results

In the 20 prospective cohorts with follow-up 8 to 26 years, 36,297 incident invasive BC cases (3879 premenopausal and 25,656 postmenopausal) were identified among 1,061,915 women (Table 1). ER receptor status was known for 71.9% of cases, PR status for 68.7% of cases (Supplementary Table 1). Of the premenopausal cases with known receptor status, the percentages with ER+, PR+, ER+ PR+, and ER− PR− status were 72%, 69%, 62%, and 21%, respectively. For postmenopausal cases, the percentages with ER+, PR+, ER+ PR+, and ER− PR− status were 82%, 70%, 68%, and 15%, respectively.

Median height ranged from 1.52 m in the Japanese JPHC1 to 1.66 m in the Swedish WLHS. Median baseline BMI ranged from 22.8 kg/m^2^ in women aged 30–49 years in WLHS to 26.2 kg/m^2^ in women aged 55–74 years in the US PLCO. Among the cohort studies with available data, median early adult BMI differed by only 1 kg/m^2^ across studies (Table 1).

### Height

**Fig. 1:**
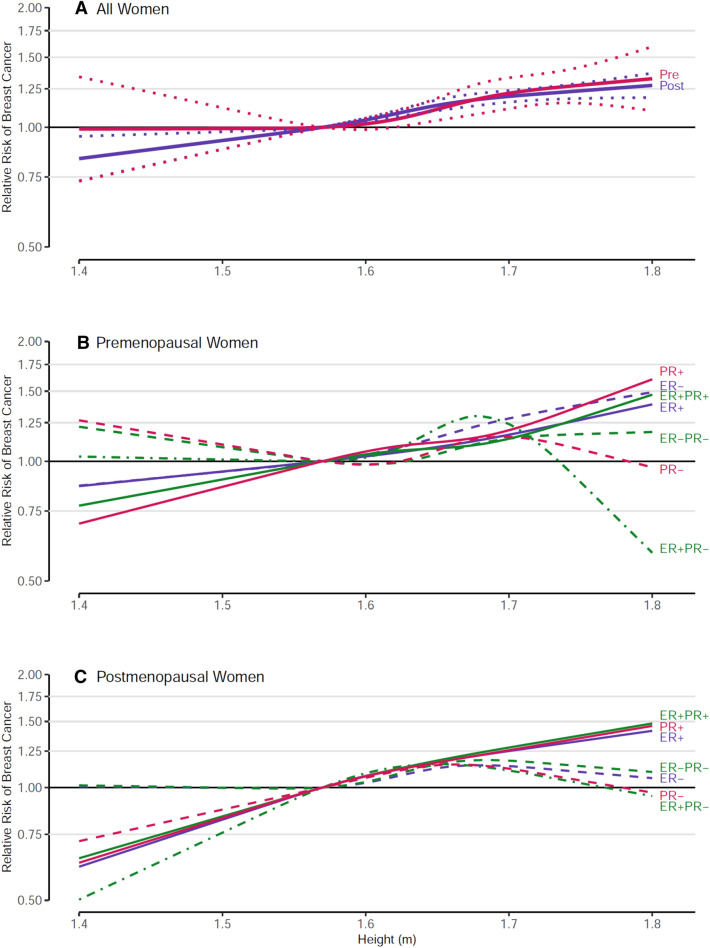
Spline regression curves for the association between height and breast cancer risk, the Pooling Project of Prospective Studies of Diet and Cancer, with reference height = 1.57 m. Panel A shows the associations for overall premenopausal breast cancer (pre (red); *p* value, test for nonlinearity = 0.363) and overall postmenopausal breast cancer (post (blue); *p* value, test for nonlinearity = 0.147). Solid lines represents point estimates and dotted lines represent 95% confidence intervals. Panel B shows the associations for premenopausal breast cancer subtypes defined by estrogen receptor (ER) and progesterone receptor (PR) status. The *p* value, test for nonlinearity = 0.846 for ER positive (ER+, blue solid line), 0.882 for ER negative (ER−, blue dashed line), 0.610 for PR+ (red solid line), 0.385 for PR− (red dashed line), 0.664 for ER+ PR+ (green solid line), 0.232 for ER+ PR− (green dash-dotted line), and 0.683 for ER− PR− breast cancer (green dashed line). Panel C shows the associations for postmenopausal breast cancer subtypes defined by ER and PR status. The *p* value, test for nonlinearity = 0.736 for ER+ (blue solid line), 0.247 for ER− (blue dashed line), 0.855 for PR+ (red solid line), 0.223 for PR− (red dashed line), 0.875 for ER+ PR+ (green solid line), 0.455 for ER+ PR− (green dash-dotted line), and 0.248 for ER− PR− breast cancer (green dashed line). Models were adjusted for ethnicity (Caucasian, African–American, Hispanic, Asian, others), family history of breast cancer (yes, no), personal history of benign breast disease (yes, no), alcohol consumption (non-drinkers, > 0–< 5, 5–< 15, 15–< 30, ≥ 30 g/day), smoking status (never, past, current), education (< high school, high school, > high school), physical activity (low, medium, high), age at menarche (< 11, 11–12, 13–14, ≥ 15 years), baseline BMI (< 23, 23–< 25, 25–< 30, ≥ 30 kg/m^2^), oral contraceptive use (never, ever), hormone replacement therapy (never, ever), energy intake (kcal/d, continuous), interaction between parity (0,1–2, ≥ 3) and age of first birth (< 30, ≥ 30 years); age at baseline in years and year of questionnaire return were included as stratification variables

Height was statistically significantly positively associated with both premenopausal and postmenopausal BC risk (Fig. 1; Supplementary Table 2). Restricted cubic spline regression analyses showed no significant deviation from linearity for these associations (*p* values, test for nonlinearity ≥ 0.14, Fig. 1). In continuous analyses, the RRs (95%CI) per 5 cm increment were similar in premenopausal and postmenopausal women: 1.07 (1.04–1.10) and 1.06 (1.05–1.08), respectively (Supplementary Table 2). There was no significant heterogeneity in RR estimates between the cohort studies, nor any significant interaction by menopausal status or by HRT use in postmenopausal women (*p* values, tests for interaction, 0.750 and 0.212, respectively).

For height, stronger positive associations were generally seen with hormone receptor-positive subtypes than with receptor-negative subtypes for both premenopausal and postmenopausal BC, except for ER+ and ER− premenopausal BC (Fig. 1, Supplementary Table 2). For postmenopausal BC, all tests for differences between subtypes defined by ER and/or PR status were significant in continuous analyses (all *p* values < 0.03, Supplementary Table 2). For both premenopausal and postmenopausal BC, relative to heights of 1.55–< 1.60 m, risk for women ≥ 1.75 m was approximately 40–50% higher for receptor-positive subtypes, but only 20% higher for receptor-negative subtypes (except a 40% increased risk was seen for premenopausal ER− subtype).

### Baseline BMI

**Fig. 2:**
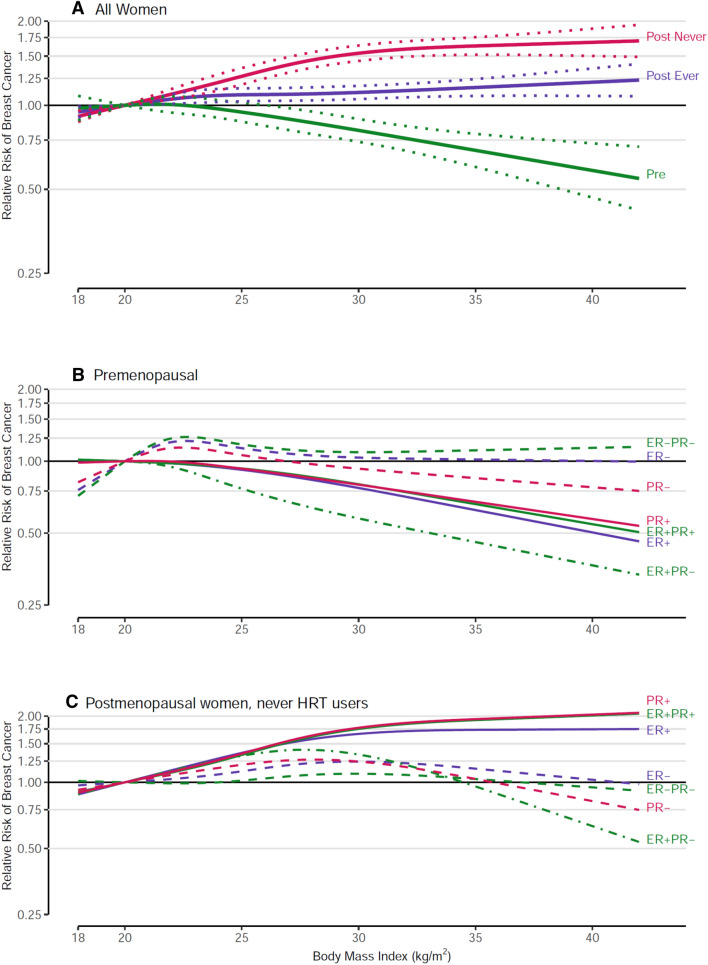
Spline regression curves for the association between body mass index at baseline and breast cancer risk, the Pooling Project of Prospective Studies of Diet and Cancer, with reference 20 kg/m^2^. Panel A shows the associations for overall premenopausal breast cancer (pre; *p* value, test for nonlinearity = 0.150), overall postmenopausal breast cancer among never users of hormone therapy (post never; *p* value, test for nonlinearity < 0.001), and overall postmenopausal breast cancer among ever users of hormone therapy (post ever; *p* value, test for nonlinearity = 0.208). Panel B shows the associations for premenopausal breast cancer subtypes defined by estrogen receptor (ER) and progesterone receptor (PR) status. The *p* value, test for nonlinearity = 0.371 for ER+, 0.043 for ER−, 0.484 for PR+, 0.111 for PR−, 0.512 for ER+ PR+, 0.911 for ER+ PR−, and 0.084 for ER− PR− breast cancer. Panel C shows the associations for postmenopausal breast cancer subtypes defined by ER and PR status among never users of hormone therapy. The *p* value, test for nonlinearity = < 0.001 for ER+, 0.138 for ER−, < 0.001 for PR+, < 0.001 for PR−, < 0.001 for ER+ PR+, < 0.001 for ER+ PR−, and 0.558 for ER− PR− breast cancer. Models were adjusted for ethnicity (Caucasian, African–American, Hispanic, Asian, others), family history of breast cancer (yes, no), personal history of benign breast disease (yes, no), alcohol consumption (non-drinkers, > 0–< 5, 5–< 15, 15–< 30, ≥ 30 g/day), smoking status (never, past, current), education (< high school, high school, > high school), physical activity (low, medium, high), age at menarche (< 11, 11–12, 13–14, ≥ 15 years), height (< 1.60, 1.60–< 1.65, 1.65–< 1.70, 1.70–< 1.75, ≥ 1.75 m), oral contraceptive use (never, ever), energy intake (kcal/d, continuous), interaction between parity (0, 1–2, ≥ 3) and age of first birth (< 30, ≥ 30 years); age at baseline in years and year of questionnaire return were included as stratification variables

Baseline (adult) BMI was strongly inversely related to premenopausal BC risk, strongly positively related to postmenopausal BC risk in HRT never-users, and only modestly positively related to postmenopausal BC risk in ever HRT users (all *p* trend < 0.001) (Fig. 2a; Supplementary Table 3), with significant interaction by menopausal status (*p* < 0.001) and HRT use (*p* < 0.001). Spline regression analyses showed a steady decline in risk with increasing baseline BMI in premenopausal women, but a plateau in increasing risk at a BMI of ~ 30 kg/m^2^ in postmenopausal never HRT users (p-nonlinearity < 0.001). Comparing women with BMI ≥ 30 kg/m^2^ to women with BMI < 21 kg/m^2^, the RR (95%CI) was 0.78 (0.64–0.93) for premenopausal BC, 1.61 (1.45–1.79) for postmenopausal BC in HRT never-users, and 1.17 (1.09–1.25) for postmenopausal HRT ever-users. There was no significant heterogeneity between the cohort studies for premenopausal BC or for postmenopausal BC in never or ever HRT users.

For premenopausal BC, the strong inverse association with baseline BMI was more pronounced for receptor-positive subtypes (all p-trend < 0.001) but flattened out and was not statistically significant for receptor-negative subtypes (Fig. 2b, Supp Table 3). Comparing BMI ≥ 30 to < 21 kg/m^2^, 32–33% significantly lower risks were observed for ER+, PR+ and ER+ PR+ subtypes. For the same contrast, the comparable RRs were not significant: 0.97 for PR−, 1.00 for ER−, and 1.19 for ER− PR− tumors.

For postmenopausal women who never used HRT, stronger positive associations with baseline BMI that plateaued around 30 kg/m^2^ were seen for receptor-positive subtypes (all p-nonlinearity < 0.001) (Fig. 2c, Supp Table 3). RRs ranged from 1.69 to 1.95 comparing BMI ≥ 30 vs. < 21 kg/m^2^ (all p-trend < 0.001). However, with receptor-negative subtypes, risk increased slightly and then declined with the comparable RRs ranging from 1.03 to 1.12 (Fig. 2c, Supp Table 3). Even with at least 1000 cases of each receptor-negative subtype, none of these RR point estimates was significant (all p for common effects by receptor status ≤ 0.002).

### Early adult BMI

**Fig. 3:**
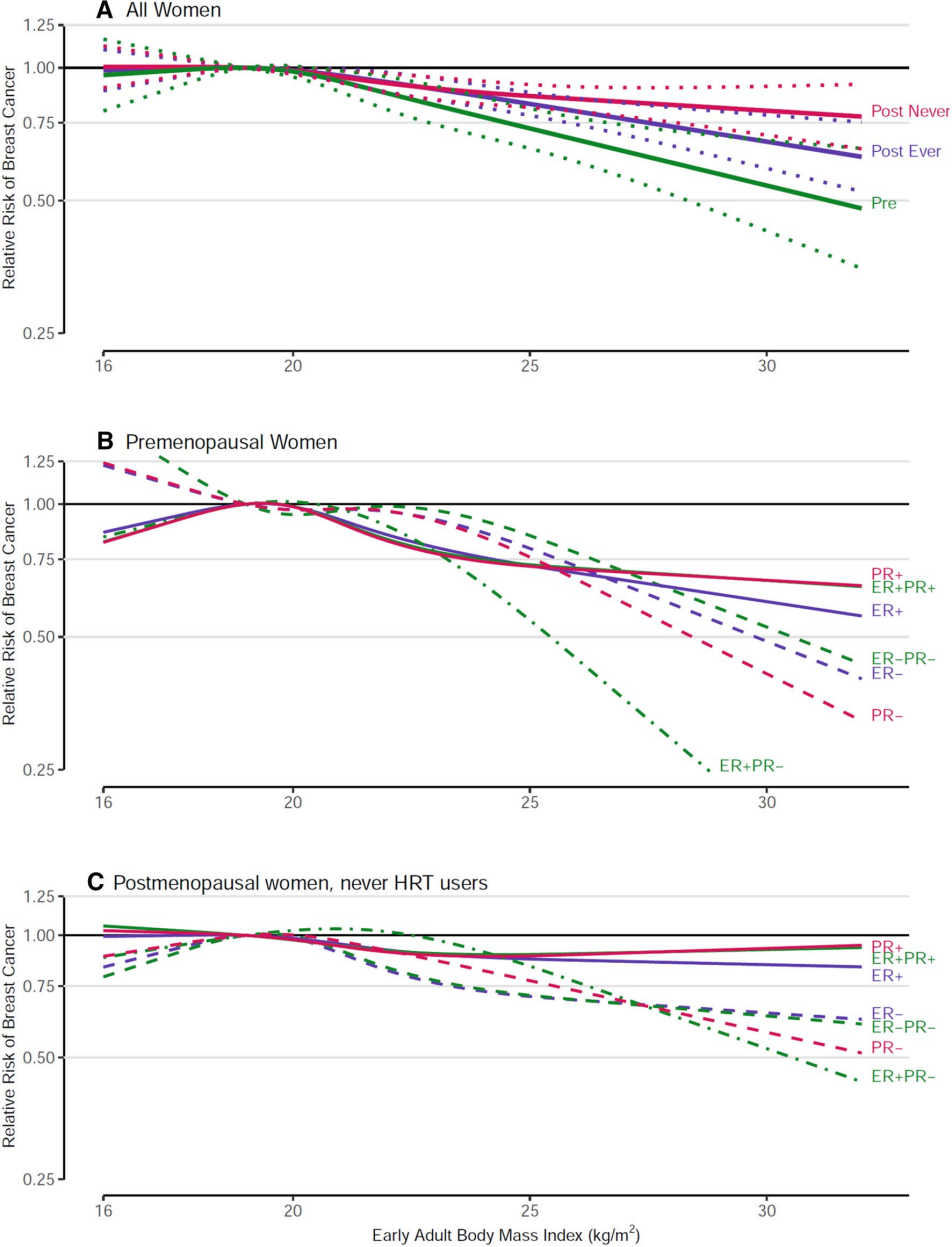
Spline regression curves for the association between early adulthood body mass index and breast cancer risk, the Pooling Project of Prospective Studies of Diet and Cancer, with reference 19 kg/m^2^. Panel A shows the associations for overall premenopausal breast cancer (pre; *p* value, test for nonlinearity = 0.088), overall postmenopausal breast cancer among never users of hormone therapy (post never; *p* value, test for nonlinearity = 0.509), and overall postmenopausal breast cancer among ever users of hormone therapy (post ever; *p* value, test for nonlinearity = 0.068). Panel B shows the associations for premenopausal breast cancer subtypes defined by estrogen receptor (ER) and progesterone receptor (PR) status. The *p* value, test for nonlinearity = 0.090 for ER+, 0.469 for ER−, 0.047 for PR+, 0.271 for PR−, 0.074 for ER+ PR+, 0.178 for ER+ PR−, and 0.347 for ER−PR− breast cancer. Panel C shows the associations for postmenopausal breast cancer subtypes defined by ER and PR status among never users of hormone therapy. The *p* value, test for nonlinearity = 0.544 for ER+, 0.130 for ER−, 0.305 for PR+, 0.074 for PR−, 0.390 for ER+ PR+, 0.060 for ER+ PR−, and 0.116 for ER−PR− breast cancer. Models were adjusted for the same factors as in Fig. 2

Early adult BMI was significantly inversely related to both premenopausal and postmenopausal BC (never and ever HRT users) risk, and associations were stronger for premenopausal BC (p-interaction by menopausal status 0.002 for continuous early adult BMI, Supplementary Table 4). In postmenopausal women, interaction with HRT use was not significant (*p* = 0.618). Restricted cubic spline analyses showed no significant deviations from linearity for premenopausal and postmenopausal BC among never HRT users and among ever HRT users (Fig. 3), overall or for most subtypes. Associations were moderately to strongly inverse with no significant heterogeneity between cohort studies. Per increment of 5 kg/m^2^, the RR (95% CI) was 0.79 (0.74–0.84) for premenopausal, and 0.89 (0.85–0.92) for postmenopausal BC in never HRT users. The inverse associations appeared somewhat stronger for receptor-negative than for receptor-positive subtypes. However, these differences were only significant for PR status for postmenopausal BC among never HRT users (p-common effects = 0.020), (Fig. 3, Supplementary Table 4).

### Adult weight change

**Fig. 4:**
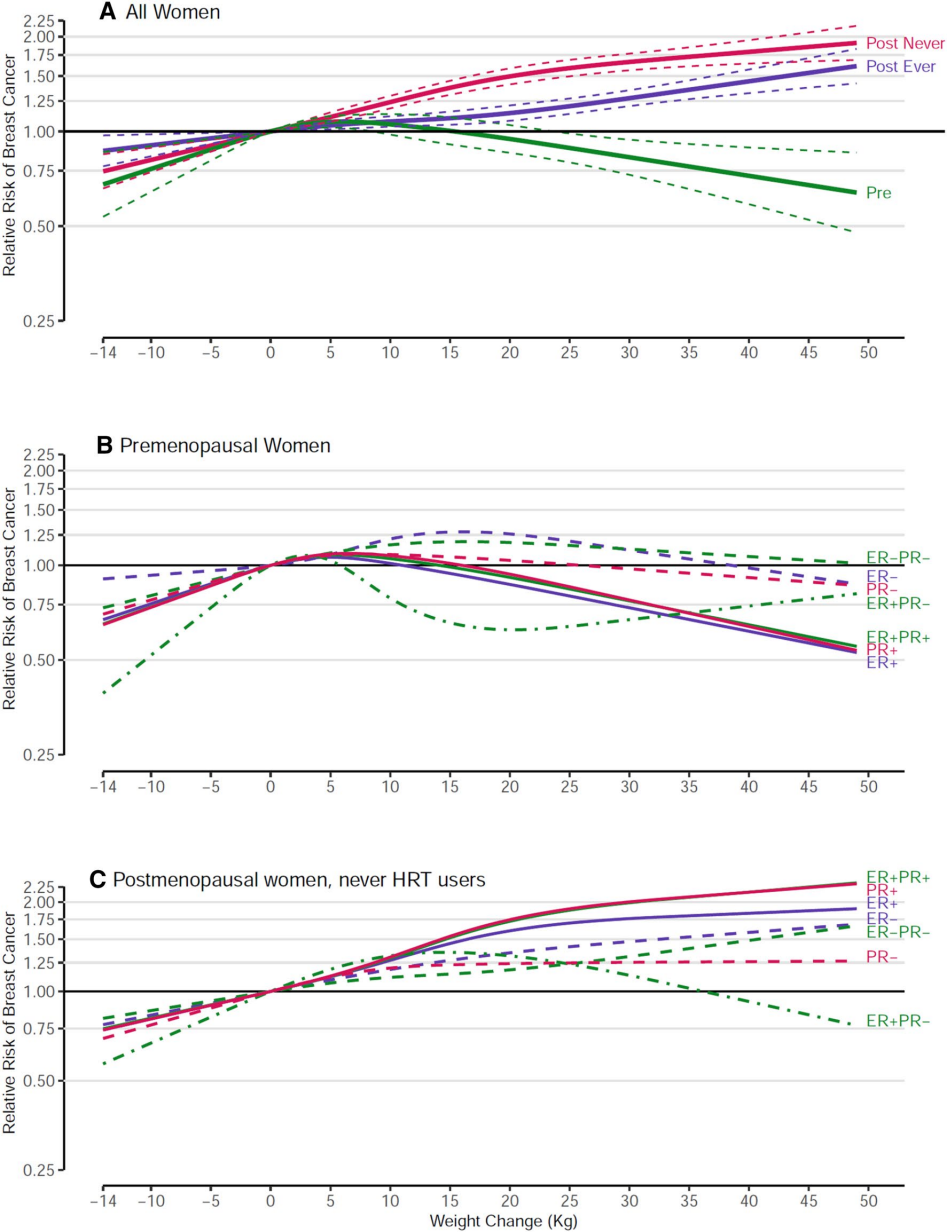
Spline regression curves for the association between weight change and breast cancer risk, the Pooling Project of Prospective Studies of Diet and Cancer, with reference 0 kg. Panel A shows the associations for overall premenopausal breast cancer (pre; *p* value, test for nonlinearity < 0.001), overall postmenopausal breast cancer among never users of hormone therapy (post never; *p* value, test for nonlinearity < 0.001), and overall postmenopausal breast cancer among ever users of hormone therapy (post ever; *p* value, test for nonlinearity = 0.279). Panel B shows the associations for premenopausal breast cancer subtypes defined by estrogen receptor (ER) and progesterone receptor (PR) status. The *p* value, test for nonlinearity < 0.001 for ER+, 0.124 for ER−, < 0.001 for PR+, 0.222 for PR−, < 0.001 for ER+ PR+, 0.059 for ER+ PR−, and 0.402 for ER− PR− breast cancer. Panel C shows the associations for postmenopausal breast cancer subtypes defined by ER and PR status among never users of hormone therapy. The *p* value, test for nonlinearity < 0.001 for ER+, 0.630 for ER−, < 0.001 for PR+, 0.036 for PR−, 0.003 for ER+ PR+, < 0.001 for ER+ PR−, and 0.874 for ER− PR− breast cancer. Models were adjusted for the same factors as in Fig. 2

Adult weight change between age 18–20 and age at study enrollment was not significantly associated with premenopausal BC risk but significantly positively associated with postmenopausal BC risk in both never and ever HRT users (both p-trend < 0.001) (Fig. 4a, Supp Table 5). Interaction by menopausal status was significant (*p* < 0.001). Postmenopausal women with a weight gain of ≥ 20 kg, compared to women with stable weight (defined as a change of 2 kg or less), had a 68% increased risk of BC in never HRT users, and a 22% increased risk in ever HRT users (p-interaction by HRT use  < 0.001). Spline regression analyses confirmed significant nonlinearity for premenopausal BC and postmenopausal BC in never HRT users, but not for postmenopausal BC in ever HRT users. In continuous analyses, the RR (95% CI) was 1.09 (1.06–1.12) per increment of 10 kg for postmenopausal BC in ever HRT users. Estimates were essentially similar when early adult BMI was included in the models (data not shown). Compared to women maintaining a stable weight, women who had lost > 2 kg had nonsignificant 9–10% lower risk of both overall premenopausal and postmenopausal BC (Supplementary Table 5).

For premenopausal BC, spline regression analyses showed evidence of nonlinear relationships with weight change for hormone receptor-positive subtypes, with risk increasing and then decreasing, and no associations with receptor-negative subtypes (Fig. 4b, Supplementary Table 5). For postmenopausal BC among never HRT users, significant positive associations with weight change were seen for all receptor subtypes (all p-trend ≤ 0.04); there was evidence for nonlinearity for nearly all subtypes (Fig. 4c, Supplementary Table 5). Positive associations were substantially stronger for receptor-positive subtypes, with an increased risk of approximately 70–90% associated with a weight gain of ≥ 20 kg, relative to stable weight, compared to 10–40% for receptor-negative subtypes.

### Joint effect of early adult BMI and adult weight change

To examine whether the association between adult weight change and subsequent BC risk depended on BMI in early adulthood, we conducted analyses of adult weight change stratified by early adult BMI (Supplementary Table 6). For these analyses, the group of women was split at early adult BMI of 21 kg/m^2^ (close to the median of BMI age 18–20 years across the cohort studies), and the common reference category was the group of women with early adult BMI < 21 kg/m^2^, and stable weight. For premenopausal BC, and its ER+ subtype, risk was decreased at ≥ 20 kg weight gain; for the ER− subtype there was essentially no association. There was no evidence of interaction between early adult BMI and weight change. Women heavier in early adulthood generally have reduced premenopausal BC risk, relative to those leaner in early adulthood, independent of adult weight change.

Postmenopausal BC risk was strongly related to weight change in both strata of early adult BMI among women who never used HRT (both p-trend < 0.001). The positive association was more pronounced for women who were leaner in early adult life (BMI18 < 21), with a 79% increased risk for those who gained ≥ 20 kg compared to women with stable weight (p-interaction = 0.018). For ER+ postmenopausal BC, the same pattern was observed with a significant interaction (p-interaction = 0.034). For both overall and ER+ BC, risk was noticeably lower (approximately 20–35% reduction) among women who were leaner in early adult life and lost > 2 kg, whereas risk was only slightly reduced (by < 10%) among women with a comparable weight loss who were heavier in early adult life. For ER− BC, weight change was only significantly positively associated in women with early adult BMI < 21 kg/m^2^ (p-trend < 0.001); no clear relationship was seen in women with early adult BMI ≥ 21 kg/m^2^ (p-interaction = 0.133).

## Discussion

Height showed positive, linear associations for premenopausal and postmenopausal BC risk, with stronger associations for hormone receptor-positive subtypes. Baseline (adult) BMI had inverse associations with premenopausal BC, with stronger associations for receptor-positive subtypes. Positive nonlinear associations between baseline BMI and postmenopausal BC risk were found in women who never used HRT, primarily for receptor-positive subtypes; positive associations were more modest in HRT users than never users. Early adult BMI was inversely related to both premenopausal and postmenopausal BC risk with suggestively stronger associations shown for receptor-negative subtypes. Adult weight gain since age 18–20 years was positively associated with postmenopausal BC risk; the association was substantially stronger for receptor positive subtypes, and among women who were leaner in early adulthood. Women who had lost weight since age 18–20 years had a suggestively lower risk of overall premenopausal or postmenopausal BC compared to women with stable weight. Women heavier in early adulthood generally had reduced premenopausal BC risk, relative to those leaner in early adulthood, independent of adult weight change.

This large pooled analysis of prospective studies provides the most detailed examination of individual height, weight and adult weight change in relation to postmenopausal BC risk overall and by hormone receptor subtype. Further strengths of this analysis include use of standardized exposure and confounder information, examination of overall premenopausal BC and receptor subtypes and use of a standardized (dose–response) modeling approach across outcomes and populations. Unlike meta-analyses of published results on these topics which have often reported heterogeneity between studies (e.g. [11, 20]), our analysis generally showed little heterogeneity between cohorts after stratifying by menopausal status, HRT use, and ER/PR subtypes. Our study enabled us to conduct more detailed dose–response modelling, particularly for BC subtypes, than was accomplished in many other studies, including meta-analyses. This is especially important when relationships are nonlinear, as we observed for baseline BMI and weight gain with postmenopausal BC risk in women who never used HRT and for weight gain with (hormone receptor-positive subtypes of) premenopausal BC.

### BMI at baseline (generally in mid to later adulthood)

While our current analysis confirms the stark contrasting associations between baseline BMI and risk of premenopausal (inverse association) versus postmenopausal BC (positive association) [1, 2, 6, 7, 56–59], and stronger positive associations with postmenopausal BC in never compared to ever HRT users [7, 56, 60], we also showed that the dose–response relationship with postmenopausal BC and its subtypes was significantly nonlinear in never HRT users. As in recent meta-analyses [19, 20], including at most half of the studies in our present analysis, we now showed relatively strong positive associations with ER and/or PR positive subtypes (ER+, PR+, ER+ PR+) of postmenopausal BC, and no statistically significant associations with receptor-negative subtypes. In contrast to meta-analyses which can often be hampered by heterogeneity in the study-specific results [7, 20, 57], we observed no significant heterogeneity among the cohorts after taking into account menopausal status and HRT use, illustrating the potential of more precise pooled analyses.

Regarding the association of overweight in later adult life with postmenopausal BC risk, the most plausible mechanism is hormone-related. When ovarian estrogen production is decreased after menopause, adipose tissue again becomes a major source of estrogen due to aromatization of androgens in peripheral adipose tissue [61]. Together with reduced production of sex hormone binding globulin due to obesity, this leads to increased levels of circulating bioavailable estrogens. Support for the mediating role of estrogens comes from observations that the association between BMI and postmenopausal BC was essentially eliminated after adjustment for bioavailable estrogen concentration [14, 15, 58]. Furthermore, the substantially weakened association with BMI in HRT users also indicates that this exogenous hormone source overrides the effect of endogenous estrogen production from peripheral fat tissue. Thus, the association of postmenopausal BC with BMI is most visible in never HRT users, and indeed specifically with hormone receptor positive subtypes. Alternative potential biological mechanisms include altered production of adipokines, excess activation of the IGF axis, and chronic low-grade inflammation [62–64].

For BMI and premenopausal BC, we found stronger inverse associations than previously reported in meta-analyses [7, 20, 57]; our results were similar to those of a recent pooled analysis focused on premenopausal BC (8 studies in our analyses overlapped with that pooled analysis) [8]. We also showed stronger inverse associations with hormone receptor-positive subtypes, and no significant association with receptor-negative subtypes of premenopausal BC, as has also been seen in meta-analyses of cohort and case–control studies combined [6, 19]. For the inverse association between BMI and premenopausal BC risk, several mechanisms have been proposed but none has been widely accepted. In premenopausal women, obesity may protect against BC by causing more frequent anovulatory menstrual cycles [65, 66]. This would result in decreased estradiol and progesterone exposure and lower luteal phase progesterone levels in ovulatory cycles [67]. Although this is not supported by studies that have adjusted for menstrual cycle pattern [68–70], our observation that BMI is more strongly related to hormone-dependent ER/PR positive BCs nevertheless suggests a hormonal mechanism is partly responsible. Other proposed mechanisms include mitigation of estrogen-induced proliferation in breast epithelial cells concentrations in premenopausal women by decreased progesterone [20]. Gene expression studies showed decreased BC cell proliferation in premenopausal women with increasing body weight, but increased in postmenopausal women [20, 71]. More research is needed to elucidate this intriguing inverse association of BMI with premenopausal BC.

### Early adult BMI

Intriguingly, BMI at early adult age (18–20 years) seems consistently inversely related to both premenopausal and postmenopausal BC risk, as is evident from our pooled analysis, and from meta-analyses of cohort studies [11, 20]. Unlike results from meta-analyses [11, 20], there was no statistically significant heterogeneity in our cohort estimates. Also, our estimated association regarding premenopausal BC (RR = 0.79 per 5 kg/m^2^) was more strongly inverse than in the meta-analyses [11, 20], and comparable to a recently published estimate from a pooled analysis of premenopausal BC [8]. This latter study showed that BMI at ages 18–24 years was most strongly inversely related to premenopausal BC, compared to BMI measured at ages 25–54 years. Although both that pooled study and our study reported no significant differences in associations across tumor subtypes defined by hormone receptor status, we noted that stronger associations were suggested for receptor-negative subtypes for both premenopausal and postmenopausal BC. For postmenopausal BC, we also observed that the inverse associations were statistically significant for all ER/PR subtypes except ER+ PR−, a subtype with relatively few cases.

A possible explanation for the intriguing inverse association of early BMI and BC was given in a Finnish study, which found that BMI at age 7 years showed a stronger inverse association with BC than BMI at age 15 [72], which is in line with the observation that BMI at ages 18–24 years had a stronger inverse relationship with BC than BMI measured at later ages [8]. BMI is an indicator of estrogenicity in childhood, especially before puberty, when adipose tissue is the major source of estrogens [72]. Mammary glands may differentiate early in animals exposed to estrogens early in life, and therefore become less susceptible to carcinogens [72–74]. Increased estrogenicity resulting from childhood overweight or obesity, especially before puberty, possibly also induces early human breast differentiation [72], and thus could possibly lead to reduced BC risk later. Other mechanisms related to IGF1 are also possible, since it has been found that higher BMI in childhood/adolescence was associated with decreased insulin-like growth factor1 (IGF1) levels in adult women [11, 75]. Because the mechanism for a protective effect of early adiposity on BC remains uncertain, more research is needed to obtain further mechanistic insight.

### Adult weight change

Our results on weight change and postmenopausal BC generally agree with results from a recent meta-analysis [20], although we found positive associations with all postmenopausal ER/PR subtypes instead of only receptor-positive subtypes [20]. After stratification by early adult BMI, we found that adult weight gain after ages 18–20 years was more important for determining postmenopausal BC risk (especially in never HRT users) than early adult BMI, with the strongest positive effect of weight gain observed in women with early adult BMI < 21 kg/m^2^. In line with these results, Song et al. [63] observed in an analysis of body shape trajectories across the lifespan and cancer risk, that women who were lean in adolescence and gained weight later in life had a significantly higher postmenopausal BC risk than stable lean women. Furthermore, women with a heavier body shape in early life showed a lower risk of total and obesity-related cancer than women with a leaner body shape, which seemed to be mainly due to postmenopausal BC [63]. For premenopausal BC, early adult BMI seemed to be more important for the inverse association than adult weight gain.

### Height

Our results showing moderately strong, positive, linear associations of height and BC risk are consistent with earlier studies and meta-analyses [1, 3–5, 41]. In a meta-analysis of 26 cohort studies, height was positively associated with premenopausal and postmenopausal BC [5]; the strength of the associations was comparable to our current results. That meta-analysis also reported stronger associations for hormone receptor positive subtypes, but did not distinguish premenopausal from postmenopausal BC. We found generally stronger associations for receptor-positive subtypes (particularly PR+) for both premenopausal and postmenopausal BC, but these differences were only statistically significant in postmenopausal BC, possibly due to lower numbers of premenopausal BCs. Some evidence suggests that height is positively associated with PR expression in normal breast tissue [76]. The association of height with BC risk was also confirmed in Mendelian randomization analysis [5], underscoring the strong and consistent evidence for height as a risk factor for BC.

Attained adult height is genetically and environmentally determined. Recent results from the Netherlands Cohort Study show that women who were exposed to energy restriction (i.e. father unemployed vs. employed) during the Economic Depression (1932–1940) before or during their growth spurt, were significantly shorter than those without this exposure (163.8 cm vs. 165.5 cm, respectively) [41]. In that study, energy restriction before and/or during the pubertal growth spurt was associated with a decreased hormone receptor-positive BC risk [41]. One mechanism as to why height increases BC risk involves the IGF signaling pathway as IGFs, and particularly IGF1, play important roles in growth and in carcinogenesis by inhibiting apoptosis and stimulating cell proliferation [5, 77–80].

### Study limitations

Besides the abovementioned strengths of our study, potential weaknesses of our study include the use of self-reported height and weight in the majority of studies, the longer-term recall of early adult weight, the lack of detailed information on menopausal status and other covariates such as HRT use during follow-up, absence of weight information at ages other than early adulthood and cohort baseline, limited number of studies from continents other than North-America and Europe, and minimal race/ethnic diversity. The group of ever HRT users was heterogeneous regarding type of HRT used (which may be important for BC [81]), and consisted of past and current users because we had no updated information on use. In a recent pooled analysis, no association between BC risk and BMI was seen among current HRT users [81]. We also had no information on the mode of BC detection, whereas associations of BMI with BC may vary according to detection method [82].

## Conclusion

In conclusion, associations between baseline (adult) BMI and adult weight change with risk of BC varied according to menopausal status, were often nonlinear, and stronger for hormone receptor-positive subtypes for both premenopausal and postmenopausal BC. Body height was linearly and positively associated with any BC risk, but stronger for receptor-positive subtypes. In contrast, the inverse and generally linear associations of early adult BMI with premenopausal and postmenopausal BC seemed stronger for receptor-negative subtypes. The association of baseline (adult) BMI with postmenopausal BC risk was stronger in never HRT users than in ever users. If HRT use further decreases in the future, then BMI will become a more important, avoidable cause of postmenopausal BC [6]. More research is needed to identify mechanisms of action for the intriguing inverse association of BMI with premenopausal BC risk and the crossover of effects of BMI on premenopausal and postmenopausal BC risk, the inverse association of early adult BMI with premenopausal and postmenopausal BC, and the positive association of height with premenopausal and postmenopausal BC risk.

## Electronic supplementary material

Below is the link to the electronic supplementary material.

Supplementary material 1 (PDF 1480 kb)
