# Supplementary material for: Body size and weight change over adulthood and risk of breast cancer by menopausal and hormone receptor status: a pooled analysis of 20 prospective cohort studies
Source: Eur J Epidemiol. 2020 Oct 30;36(1):37–55. doi: 10.1007/s10654-020-00688-3 (PMC7847460; doi:10.1007/s10654-020-00688-3)
Supplement: Supplementary file 1 — Supplementary material 1 (PDF 1480 kb) [file 10654_2020_688_MOESM1_ESM.pdf]

SUPPLEMENTARY MATERIALS to:

P.A. van den Brandt et al. "Body size and weight change over adulthood and risk of breast cancer by menopausal and hormone receptor status: a pooled analysis of 20 prospective cohort studies"

European Journal of Epidemiology 2020

Supplementary Table S1. Number of breast cancer cases and hormone receptor subtypes in the cohort studies included in the pooled analyses of anthropometry and breast cancer risk by estrogen receptor (ER) and progesterone receptor (PR) status, Pooling Project of Prospective Studies of Diet and Cancer.

Supplementary Table S2. Pooled multivariable-adjusted RRs with 95% CIs of pre- and postmenopausal breast cancer for height, by hormone receptor subtype.

Supplementary Table S3. Pooled multivariable-adjusted RRs with 95% CIs of pre- and postmenopausal breast cancer for baseline BMI, by hormone receptor subtype.

Supplementary Table S4. Pooled multivariable-adjusted RRs with 95% CIs of pre- and postmenopausal breast cancer for early adult BMI (at age 18-20 years) by hormone receptor subtype early adult BMI (at age 18-20 years).

Supplementary Table S5. Pooled multivariable-adjusted RRs with 95% CIs of pre- and postmenopausal breast cancer for adult weight change between age 18-20 years and cohort baseline, by hormone receptor subtype early adult BMI (at age 18-20 years).

Supplementary Table S6. Pooled multivariable-adjusted RRs with 95% CIs of pre- and postmenopausal breast cancer for adult weight change between age 18-20 years and cohort baseline, stratified by BMI at age 18-20 years.

Supplementary Table S7. Funding and acknowledgements for individual cohorts.

Table S1. Number of breast cancer cases and hormone receptor subtypes in the cohort studies included in the pooled analyses of anthropometry and breast cancer risk by estrogen receptor (ER) and progesterone receptor (PR) status, Pooling Project of Prospective Studies of Diet and Cancer

Study (country)	Acronym	Baseline cohort size*	Years of follow-up	Breast cancer cases								
				Total	ER+	ER-	PR+	PR-	ER+PR+	ER+PR-	ER-PR+	ER-PR-
Beta-Carotene and Retinol Efficacy Trial (USA)	CARET	5,939	1985-2005	363	190	31	161	47	158	21	3	26
Breast Cancer Detection Demonstration Project Follow-up Study (USA)	BCDDP	38,846	1987-1999	1219	747	157	631	254	600	127	30	124
California Teachers Study (USA)	CTS	96,416	1995-2003	2579	1848	327	1472	600	1441	303	28	296
Canadian National Breast Screening Study (Canada)	CNBSS	44,671	1980-2000	1228	362	125	305	139	268	47	21	76
Cancer Prevention Study II Nutrition Cohort (USA)	CPSII	72,999	1992-2003	2952	1805	320	1454	555	1413	279	36	270
CLUE II : Campaign Against Cancer and Heart Disease (USA)	CLUE II	8,263	1989-2007	287	198	49	168	77	159	37	9	40
Iowa Women's Health Study (USA)	IWHS	34,536	1986-2004	1848	1328	238	1117	387	1082	190	34	196
Japan Public Health Center-Based Prospective Study 1 (Japan)	JPHC1	21,468	1990-2004	288	111	69	87	82	74	30	13	52
Melbourne Collaborative Cohort Study (Australia)	MCCS	22,429	1990-2006	799	493	171	420	240	393	96	26	144
Multiethnic Cohort (USA)	MEC	90,394	1993-2004	3261	2129	539	1738	760	1669	300	69	460
Netherlands Cohort Study (Netherlands)	NLCS	62,573	1986-1999	1959	679	177	351	188	338	94	13	92
New York University Women's Health Study (USA)	NYUWHS	13,147	1985-2003	912	388	120	293	202	269	110	24	91
NIH-AARP Diet and Health Study (USA)	NIH-AARP	192,423	1995-2003	5768	2241	448	1850	759	1788	368	54	390
Nurses' Health Study (a) (USA)	NHSa	88,024	1980-1986	1113	523	254	387	302	343	103	33	185
Nurses' Health Study (b) (USA) †	NHSb	66,814	1986-2006	4358	2995	742	2410	1251	2296	582	95	626
Nurses' Health Study II (USA)	NHS II	90,922	1991-2003	1289	821	292	743	355	689	115	48	229
Hormones and Diet in the Etiology of Breast Cancer (Italy)	ORDET	8,958	1987-2002	280	204	66	178	91	157	46	19	45
Prostate, Lung, Colorectal, and Ovarian Cancer Screening Trial (USA)	PLCO	27,992	1993-2007	1082	850	137	751	226	743	100	8	126
Swedish Mammography Cohort (Sweden)	SMC	58,668	1987-2005	2508	1549	371	1257	654	1151	386	103	266
Women's Health Study (USA)	WHS	37,568	1993-2004	1161	927	182	808	284	786	124	22	160
Women's Lifestyle and Health Study (Sweden)	WLHS	45,679	1991-2006	1043	715	189	593	300	554	150	39	149
Total		1,061,915		36,297	21,103	5,004	17,174	7,753	16,371	3,608	727	4,043

*Cohort size after applying study-specific exclusion criteria and then excluding women with previous cancer diagnosis (other than nonmelanoma skin cancer); the Netherlands Cohort Study was analyzed as a case-cohort study and the above exclusions were not applied to its baseline cohort size.

† Nurses' Health Study (b) is not included as part of total cohort size because they are included in Nurses' Health Study (a). See Methods for further explanation.

Table S2. Pooled multivariable-adjusted relative risks (RRs)* with (95% confidence intervals, CIs) of breast cancer for height by estrogen receptor (ER) and progesterone receptor (PR) status, in the Pooling Project of Prospective Studies of Diet and Cancer

		Category of height (m)- RRs (95%CIs)									Continuous height (per 5 cm)		
**Breast cancer subtype**	**Number of cases**	**< 1.55**	**1.55-<1.60**	**1.60-<1.65**	**1.65-<1.70**	**1.70-<1.75**	**≥1.75**	***P*-value, test for trend**	**P-value, test for between-studies heterogeneity, highest category**	**P-value, test for common effects by receptor status, highest category**	**RRs (95%CI)**	***P*-value, test for between-studies heterogeneity**	***P*-value, test for common effects by hormone receptor status**
**Premenopausal**													
Overall	3845	1.04 (0.90-1.21)	1	1.04 (0.93-1.17)	1.16 (1.03-1.31)	1.24 (1.09-1.41)	1.33 (1.12-1.59)¶	<0.001	0.370		1.07 (1.04-1.10)¶	0.393	
ER+	2124	1.05 (0.85-1.30)	1	1.07 (0.91-1.25)	1.13 (0.96-1.32)	1.24 (1.04-1.48)	1.37 (1.10-1.70)	0.062	0.850		1.05 (1.01-1.10)	0.154	
ER-	833	1.06 (0.74-1.53)	1	1.12 (0.84-1.50)	1.28 (0.99-1.65)	1.33 (1.00-1.77)	1.44 (1.00-2.08)	0.005	0.476	0.812†	1.08 (1.02-1.14)	0.449	0.470†
PR+	2000	1.09 (0.87-1.36)	1	1.15 (0.97-1.36)	1.20 (1.02-1.42)	1.29 (1.07-1.55)	1.52 (1.22-1.90)	0.012	0.770		1.07 (1.03-1.12)	0.214	
PR-	830	1.16 (0.84-1.59)	1	1.01 (0.79-1.31)	1.15 (0.89-1.48)	1.22 (0.93-1.60)	1.16 (0.81-1.68)	0.312	0.978	0.220‡	1.03 (0.97-1.09)	0.374	0.004‡
ER+PR+	1741	1.09 (0.85-1.41)	1	1.11 (0.93-1.33)	1.15 (0.96-1.37)	1.22 (1.00-1.48)	1.41 (1.11-1.78)	0.011	0.948		1.06 (1.02-1.11)	0.390	
ER+PR-	190	1.26 (0.42-3.74)	1	1.09 (0.62-1.90)	1.15 (0.59-2.22)	1.08 (0.60-1.93)	1.07 (0.47-2.39)	0.859	0.525		1.00 (0.90-1.12)	0.712	
ER-PR-	513	1.11 (0.74-1.65)	1	0.97 (0.70-1.34)	1.15 (0.84-1.58)	1.15 (0.80-1.63)	1.23 (0.77-1.97)	0.498	0.589	0.741§	1.03 (0.93-1.14)	0.080	0.595§
**Postmenopausal**													
Overall	25618	0.94 (0.88-1.00)	1	1.07 (1.03-1.12)	1.14 (1.09-1.21)	1.20 (1.14-1.26)	1.27 (1.18-1.36)**	<0.001	0.802		1.06 (1.05-1.08)**	0.313	
ER+	14792	0.93 (0.86-1.01)	1	1.09 (1.02-1.17)	1.17 (1.08-1.26)	1.24 (1.14-1.34)	1.36 (1.24-1.48)	<0.001	0.843		1.08 (1.06-1.09)	0.420	
ER-	3137	0.96 (0.82-1.12)	1	1.03 (0.90-1.17)	1.10 (0.95-1.27)	1.17 (1.01-1.34)	1.22 (0.95-1.56)	0.012	0.218	0.431†	1.04 (1.01-1.07)	0.861	0.024†
PR+	11892	0.93 (0.85-1.02)	1	1.09 (1.02-1.17)	1.17 (1.07-1.27)	1.28 (1.18-1.38)	1.40 (1.27-1.54)	<0.001	0.883		1.09 (1.07-1.10)	0.495	
PR-	5065	0.93 (0.83-1.04)	1	1.04 (0.93-1.16)	1.12 (0.99-1.25)	1.12 (1.00-1.26)	1.18 (1.01-1.38)	0.001	0.478	0.075‡	1.04 (1.02-1.06)	0.865	0.001‡
ER+PR+	11462	0.94 (0.85-1.03)	1	1.10 (1.02-1.19)	1.17 (1.09-1.27)	1.28 (1.18-1.39)	1.42 (1.29-1.57)	<0.001	0.644		1.09 (1.07-1.10)	0.711	
ER+PR-	2374	0.89 (0.76-1.05)	1	1.07 (0.93-1.22)	1.13 (0.97-1.32)	1.05 (0.89-1.24)	1.23 (0.98-1.54)	0.011	0.634		1.04 (1.01-1.08)	0.896	
ER-PR-	2547	0.97 (0.80-1.17)	1	1.06 (0.89-1.26)	1.10 (0.92-1.32)	1.21 (1.03-1.41)	1.23 (0.93-1.63)	0.013	0.221	0.367§	1.04 (1.01-1.07)	0.637	0.006§

* Adjusted for ethnicity (Caucasian, African-American, Hispanic, Asian, others), family history of breast cancer (yes, no), personal history of benign breast disease (yes, no), alcohol consumption (non-drinkers, >0-<5, 5-<15, 15-<30, ≥30 g/d), smoking status (never, past, current), education (<high school, high school, >high school), physical activity (low, medium, high), age at menarche (<11, 11-12, 13-14, ≥15 yrs), baseline BMI (<23, 23-<25, 25-<30, ≥30 kg/m^2), oral contraceptive use (never, ever), hormone replacement therapy (never, ever), energy intake (kcal/d, continuous), interaction between parity (0,1-2, ≥3) and age of first birth (<30, ≥ 30 yrs); age at baseline in years and year of questionnaire return were included as stratification variables.

† P-value for test for differences between ER- and ER+ subtypes.
‡ P-value for test for differences between PR- and PR+ subtypes.
§ P-value for test for differences between ER+PR+, ER+PR-, and ER-PR- subtypes (ER-PR+ had insufficient case numbers).
¶ P-value for test for interaction by menopausal status, categorical exposure: 0.673; continuous: 0.750.
** P-value for test for interaction by HRT use, categorical exposure: 0.994; continuous: 0.212.

Table S3. Pooled multivariable-adjusted relative risks (RRs)* with (95% confidence intervals, CIs) of breast cancer for baseline BMI by estrogen receptor (ER) and progesterone receptor (PR) status, in the Pooling Project of Prospective Studies of Diet and Cancer.

Breast cancer subtype	Number of cases	Category of baseline BMI (kg/m^2)- RRs (95%CIs)									Continuous baseline BMI (per 5 kg/m^2)		
		< 21	21-<23	23-<25	25-<27	27-<30	≥30	*P* -value, test for trend	P-value, test for between-studies heterogeneity, highest category	P-value, test for common effects by receptor status, highest category	RRs (95%CIs)	*P* -value, test for between-studies heterogeneity	*P* -value, test for common effects by hormone receptor status
**Premenopausal**													
Overall	3845	1	1.06 (0.95-1.18)	0.95 (0.84-1.08)	0.95 (0.85-1.07)	0.89 (0.79-1.01)	0.78 (0.64-0.93)**	<0.001	0.100		0.89 (0.85-0.92)**	0.711	
ER+	2124	1	1.02 (0.90-1.16)	0.86 (0.73-1.02)	0.94 (0.81-1.10)	0.90 (0.76-1.06)	0.67 (0.53-0.84)	<0.001	0.195		0.88 (0.83-0.92)	0.491	
ER-	833	1	1.15 (0.94-1.41)	1.17 (0.94-1.45)	1.10 (0.85-1.42)	1.04 (0.78-1.39)	1.00 (0.75-1.34)	0.438	0.611	0.031†	¶		
PR+	2000	1	1.04 (0.92-1.17)	0.90 (0.75-1.07)	0.98 (0.84-1.15)	0.92 (0.77-1.09)	0.68 (0.57-0.82)	<0.001	0.793		0.88 (0.84-0.93)	0.794	
PR-	830	1	1.14 (0.94-1.39)	1.09 (0.75-1.59)	1.04 (0.80-1.36)	0.94 (0.71-1.25)	0.97 (0.65-1.46)	0.175	0.131	0.896‡	0.93 (0.86-1.02)	0.383	0.288‡
ER+PR+	1741	1	1.00 (0.88-1.15)	0.86 (0.74-0.99)	0.97 (0.82-1.14)	0.90 (0.75-1.08)	0.68 (0.56-0.83)	<0.001	0.585		0.88 (0.83-0.93)	0.825	
ER+PR-	190	1	1.15 (0.79-1.68)	0.59 (0.36-0.95)	1.02 (0.52-1.99)	0.70 (0.38- 1.30)	0.77 (0.40-1.47)	0.115	0.698		0.88 (0.70-1.09)	0.240	
ER-PR-	513	1	1.10 (0.85-1.42)	1.29 (0.80-2.08)	1.11 (0.80-1.54)	0.99 (0.68-1.43)	1.19 (0.69-2.07)	0.885	0.079	0.163§	0.98 (0.88-1.08)	0.393	0.231§
**Postmenopausal. never HRT users**													
Overall	11334	1	1.15 (1.06-1.25)	1.23 (1.13-1.35)	1.37 (1.26-1.49)	1.58 (1.46-1.72)	1.61 (1.45-1.79)††	<0.001	0.156		¶		
ER+	6148	1	1.15 (1.03-1.29)	1.29 (1.16-1.44)	1.43 (1.28-1.59)	1.65 (1.46-1.85)	1.69 (1.50-1.90)	<0.001	0.367		¶		
ER-	1319	1	1.29 (0.97-1.71)	1.06 (0.81-1.38)	1.34 (1.00-1.80)	1.40 (1.04-1.89)	1.12 (0.88-1.42)	0.174	0.502	0.002†	1.04 (0.98-1.10)	0.769	
PR+	4727	1	1.14 (1.00-1.29)	1.34 (1.19-1.52)	1.45 (1.28-1.64)	1.74 (1.54-1.97)	1.95 (1.72-2.21)	<0.001	0.723		¶		
PR-	2278	1	1.23 (1.04-1.45)	1.10 (0.93-1.30)	1.34 (1.13-1.58)	1.40 (1.19-1.66)	1.07 (0.89-1.28)	0.464	0.841	<0.001‡	¶		
ER+PR+	4549	1	1.14 (0.98-1.33)	1.31 (1.15-1.49)	1.44 (1.24-1.67)	1.67 (1.42-1.98)	1.90 (1.67-2.15)	<0.001	0.448		¶		
ER+PR-	1023	1	1.17 (0.91-1.49)	1.22 (0.96-1.56)	1.33 (1.04-1.71)	1.53 (1.19-1.96)	1.05 (0.80-1.37)	0.561	0.971		¶		
ER-PR-	1081	1	1.28 (0.95-1.73)	0.99 (0.73-1.35)	1.24 (0.97-1.59)	1.27 (0.96-1.67)	1.03 (0.80-1.33)	0.778	0.878	<0.001§	1.01 (0.95-1.08)	0.867	
**Postmenopausal, ever HRT users**													
Overall	12836	1	1.05 (0.97-1.13)	1.10 (1.01-1.19)	1.09 (1.00-1.19)	1.14 (1.05-1.24)	1.17 (1.09-1.25)	<0.001	0.421		1.05 (1.02-1.08)	0.122	
ER+	7932	1	1.00 (0.92-1.09)	1.07 (0.97-1.18)	1.06 (0.95-1.17)	1.12 (1.01-1.24)	1.16 (1.06-1.27)	<0.001	0.383		1.05 (1.02-1.08)	0.309	
ER-	1473	1	0.95 (0.79-1.15)	1.19 (0.99-1.43)	1.12 (0.90-1.39)	1.19 (0.98-1.45)	1.13 (0.92-1.39)	0.093	0.982	0.822†	1.06 (1.00-1.12)	0.800	0.882†
PR+	6543	1	0.98 (0.88-1.09)	1.11 (0.98-1.25)	1.07 (0.96-1.20)	1.12 (1.02-1.23)	1.24 (1.10-1.40)	<0.001	0.173		1.08 (1.05-1.11)	0.437	
PR-	2470	1	1.04 (0.90-1.19)	1.08 (0.94-1.24)	1.05 (0.90-1.21)	1.09 (0.93-1.26)	0.97 (0.82-1.15)	0.718	0.417	0.021‡	0.99 (0.94-1.04)	0.315	0.006‡
ER+PR+	6328	1	0.98 (0.87-1.10)	1.10 (0.97-1.25)	1.07 (0.94-1.22)	1.12 (1.01-1.24)	1.24 (1.10-1.40)	<0.001	0.183		1.08 (1.05-1.11)	0.437	
ER+PR-	1152	1	1.13 (0.93-1.38)	1.02 (0.83-1.25)	1.03 (0.83-1.27)	0.99 (0.79-1.24)	0.85 (0.66-1.08)	0.038	0.810		0.91 (0.85-0.98)	0.823	
ER-PR-	1216	1	0.97 (0.79-1.20)	1.12 (0.89-1.42)	1.13 (0.91-1.40)	1.22 (0.98-1.53)	1.16 (0.92-1.46)	0.239	0.971	0.023§	1.06 (1.00-1.13)	0.405	< 0.001§

* Adjusted for ethnicity (Caucasian, African-American, Hispanic, Asian, others), family history of breast cancer (yes, no), personal history of benign breast disease (yes, no), alcohol consumption (non-drinkers, >0-<5, 5-<15, 15-<30, ≥30 g/d), smoking status (never, past, current), education (<high school, high school, >high school), physical activity (low, medium, high), age at menarche (<11, 11-12, 13-14, ≥15 yrs), height (<1.60, 1.60-<1.65, 1.65-<1.70, 1.70-<1.75, ≥1.75 m), oral contraceptive use (never, ever), energy intake (kcal/d, continuous), interaction between parity (0,1-2, ≥3) and age of first birth (<30, ≥ 30 yrs); age at baseline in years and year of questionnaire return were included as stratification variables.

† P-value for test for differences between ER- and ER+ subtypes.
‡ P-value for test for differences between PR- and PR+ subtypes.
§ P-value for test for differences between ER+PR+, ER+PR-, and ER-PR- subtypes (ER-PR+ had insufficient case numbers).
¶ Continuous estimates not shown because of significant nonlinearity in splines regression.
** P-value for test for interaction by menopausal status, categorical exposure: < 0.001; continuous: < 0.001.
†† P-value for test for interaction by HRT use, categorical exposure: < 0.001.

Table S4. Pooled multivariable-adjusted relative risks (RRs)* with (95% confidence intervals, CIs) of breast cancer for early adult BMI (at age 18-20 years) by estrogen receptor (ER) and progesterone receptor (PR) status, in the Pooling Project of Prospective Studies of Diet and Cancer.

		Category of early adult BMI (kg/m^2)- RRs (95%CIs)								Continuous early adult BMI (per 5 kg/m^2)		
Breast cancer subtype	Number of cases	< 18.5	18.5-<21	21-<23	23-<25	≥25	*P*-value, test for trend	P-value, test for between-studies heterogeneity, highest category	P-value, test for common effects by receptor status, highest category	RRs (95%CIs)	*P*-value, test for between-studies heterogeneity	P-value, test for common effects by receptor status
**Premenopausal**												
Overall	3132	1.02 (0.92-1.13)	1	0.88 (0.81-0.97)	0.82 (0.72-0.93)	0.62 (0.53-0.72)**	<0.001	0.574		0.79 (0.74-0.84)**	0.446	
ER+	1786	1.03 (0.90-1.18)	1	0.87 (0.76-1.00)	0.86 (0.72-1.01)	0.67 (0.54-0.82)	<0.001	0.406		0.83 (0.76-0.90)	0.589	
ER-	667	1.01 (0.81-1.27)	1	0.85 (0.70-1.05)	0.94 (0.73-1.22)	0.65 (0.47-0.92)	0.010	0.446	0.917†	0.78 (0.68-0.90)	0.576	0.517†
PR+	1653	0.99 (0.86-1.14)	1	0.87 (0.75-1.02)	0.82 (0.69-0.98)	0.69 (0.54-0.88)	<0.001	0.306		¶		
PR-	709	1.08 (0.87-1.33)	1	0.89 (0.73-1.08)	0.98 (0.76-1.26)	0.60 (0.43-0.84)	0.003	0.595	0.510‡	0.79 (0.67-0.93)	0.294	
ER+PR+	1476	1.01 (0.87-1.17)	1	0.86 (0.70-1.06)	0.82 (0.67-0.99)	0.72 (0.55-0.92)	<0.001	0.306		0.84 (0.76-0.92)	0.493	
ER+PR-	188	1.00 (0.53-1.87)	1	0.80 (0.54-1.17)	0.66 (0.31-1.44)	0.60 (0.28-1.29)	0.059	0.792		0.79 (0.59-1.06)	0.309	
ER-PR-	449	1.11 (0.85-1.46)	1	0.92 (0.72-1.18)	1.03 (0.75-1.42)	0.64 (0.43-0.97)	0.023	0.690	0.854§	0.79 (0.62-1.01)	0.130	0.875§
**Postmenopausal, never HRT users**												
Overall	8978	1.03 (0.96-1.11)	1	0.96 (0.91-1.02)	0.89 (0.81-0.99)	0.83 (0.76-0.90)††	<0.001	0.829		0.89 (0.85-0.92)††	0.770	
ER+	4985	1.06 (0.97-1.15)	1	0.98 (0.91-1.05)	0.91 (0.80-1.04)	0.89 (0.80-1.00)	0.001	0.579		0.91 (0.87-0.96)	0.792	
ER-	1098	0.98 (0.82-1.17)	1	0.84 (0.72-0.98)	0.75 (0.57-0.97)	0.71 (0.55-0.92)	<0.001	0.518	0.117†	0.80 (0.70-0.91)	0.178	0.066†
PR+	3795	1.07 (0.98-1.18)	1	0.99 (0.91-1.07)	0.92 (0.80-1.07)	0.94 (0.83-1.06)	0.021	0.848		0.93 (0.88-0.99)	0.620	
PR-	1829	0.96 (0.84-1.11)	1	0.94 (0.84-1.06)	0.76 (0.64-0.90)	0.71 (0.58-0.87)	0.001	0.444	0.020‡	0.84 (0.75-0.93)	0.206	0.072‡
ER+PR+	3633	1.08 (0.98-1.19)	1	1.00 (0.92-1.09)	0.93 (0.79-1.08)	0.94 (0.83-1.07)	0.034	0.871		0.93 (0.88-0.99)	0.942	
ER+PR-	852	0.96 (0.78-1.18)	1	1.03 (0.87-1.22)	0.86 (0.68-1.09)	0.74 (0.50-1.10)	0.129	0.128		0.87 (0.77-0.99)	0.495	
ER-PR-	906	0.94 (0.78-1.15)	1	0.85 (0.72-1.00)	0.73 (0.53-1.00)	0.70 (0.52-0.93)	0.010	0.426	0.120§	0.81 (0.69-0.95)	0.097	0.235§
**Postmenopausal, ever HRT users**												
Overall	9884	0.98 (0.92-1.05)	1	0.94 (0.88-1.00)	0.84 (0.77-0.90)	0.78 (0.72-0.86)	<0.001	0.469		0.88 (0.84-0.92)	0.337	
ER+	6194	0.97 (0.90-1.04)	1	0.96 (0.90-1.02)	0.85 (0.77-0.94)	0.80 (0.71-0.89)	<0.001	0.690		¶		
ER-	1135	1.03 (0.85-1.25)	1	0.83 (0.71-0.96)	0.70 (0.55-0.89)	0.79 (0.54-1.14)	0.026	0.083	0.950†	0.80 (0.66-0.98)	0.010	
PR+	5064	1.00 (0.93-1.09)	1	0.96 (0.87-1.05)	0.86 (0.77-0.95)	0.83 (0.73-0.95)	0.001	0.421		0.89 (0.84-0.95)	0.270	
PR-	1905	0.91 (0.77-1.07)	1	0.87 (0.78-0.98)	0.67 (0.56-0.81)	0.69 (0.56-0.86)	0.001	0.491	0.136‡	¶		
ER+PR+	4912	1.00 (0.92-1.08)	1	0.96 (0.87-1.06)	0.85 (0.77-0.95)	0.82 (0.72-0.95)	0.001	0.358		0.89 (0.84-0.95)	0.256	
ER+PR-	894	0.81 (0.64-1.02)	1	0.91 (0.77-1.07)	0.70 (0.54-0.92)	0.65 (0.47-0.90)	0.035	0.971		¶		
ER-PR-	959	1.01 (0.81-1.27)	1	0.83 (0.71-0.98)	0.68 (0.52-0.88)	0.72 (0.47-1.12)	0.031	0.074	0.382§	1.10 (1.03-1.18)	0.140	

* Adjusted for ethnicity (Caucasian, African-American, Hispanic, Asian, others), family history of breast cancer (yes, no), personal history of benign breast disease (yes, no), alcohol consumption (non-drinkers, >0-<5, 5-<15, 15-<30, ≥30 g/d), smoking status (never, past, current), education (<high school, high school, >high school), physical activity (low, medium, high, missing), age at menarche (<11, 11-12, 13-14, ≥15 yrs), height (<1.60, 1.60-<1.65, 1.65-<1.70, 1.70-<1.75, ≥1.75 m), oral contraceptive use (never, ever), energy intake (kcal/d, continuous), interaction between parity (0,1-2, ≥3) and age of first birth (<30, ≥ 30 yrs); age at baseline in years and year of questionnaire return were included as stratification variables.

† P-value for test for differences between ER- and ER+ subtypes.
‡ P-value for test for differences between PR- and PR+ subtypes.
§ P-value for test for differences between ER+PR+, ER+PR-, and ER-PR- subtypes (ER-PR+ had insufficient case numbers).
¶ Continuous estimates not shown because of significant nonlinearity in splines regression.
** P-value for test for interaction by menopausal status, categorical exposure: 0.002; continuous: 0.002.
†† P-value for test for interaction by HRT use, categorical exposure: 0.329; continuous: 0.618.

Table S5. Pooled multivariable-adjusted relative risks (RRs)* with (95% confidence intervals, CIs) of breast cancer for adult weight change between age 18-20 years and cohort baseline, by estrogen receptor (ER) and progesterone receptor (PR) status, in the Pooling Project of Prospective Studies of Diet and Cancer.

		Category of adult weight change (kg) - RRs (95%CIs)								Continuous adult weight change (per 10 kg)		
Breast cancer subtype	Number of cases	< -2	-2 -< 2	2-<10	10-<20	≥20	*P*-value, test for trend	P-value, test for between-studies heterogeneity, highest category	*P*-value, test for common effects by hormone receptor status, highest category	RRs (95%CI)	*P*-value, test for between-studies heterogeneity	*P*-value, test for common effects by hormone receptor status
**Premenopausal**												
Overall	3132	0.90 (0.77-1.05)	1	1.05(0.93-1.19)	1.07(0.94-1.22)	0.85 (0.69-1.05)**	0.269	0.166		¶		
ER+	1786	0.89 (0.72-1.09)	1	1.01 (0.87-1.18)	1.01 (0.85-1.20)	0.76 (0.57-1.01)	0.208	0.199		¶		
ER-	667	1.05 (0.73-1.49)	1	1.09 (0.83-1.43)	1.25 (0.94-1.67)	1.07 (0.76-1.50)	0.556	0.818	0.131†	1.05 (0.98-1.13)	0.591	
PR+	1653	0.88(0.71-1.09)	1	1.05(0.89-1.23)	1.04(0.87-1.24)	0.79 (0.63-0.98)	0.196	0.730		¶		
PR-	709	0.92 (0.66-1.29)	1	1.06 (0.82-1.37)	1.16 (0.88-1.53)	0.90 (0.65-1.26)	0.922	0.661	0.501‡	1.04 (0.97-1.11)	0.678	
ER+PR+	1476	0.90 (0.71-1.13)	1	1.05(0.88-1.25)	1.03 (0.86-1.25)	0.79 (0.61-1.03)	0.214	0.339		¶		
ER+PR-	188	0.67 (0.35-1.27)	1	0.93 (0.44-1.97)	1.05 (0.58-1.88)	0.45 (0.19-1.09)	0.287	0.321		0.98 (0.85-1.12)	0.427	
ER-PR-	449	1.13 (0.73-1.75)	1	1.29 (0.92-1.80)	1.25 (0.86-1.80)	1.22 (0.80-1.86)	0.646	0.649	0.045§	1.07 (0.95-1.21)	0.135	
**Postmenopausal, never HRT users**												
Overall	8978	0.90 (0.75-1.06)	1	1.15(1.03-1.28)	1.42 (1.26-1.59)	1.68(1.48-1.90)††	< 0.001	0.256		¶		
ER+	4985	0.86 (0.69-1.09)	1	1.09 (0.95-1.25)	1.38(1.20-1.58)	1.65 (1.42-1.91)	< 0.001	0.385		¶		
ER-	1098	0.89 (0.56-1.43)	1	1.00 (0.74-1.35)	1.28 (0.97-1.70)	1.35 (1.01-1.81)	0.001	0.657	0.238†	1.10 (1.05-1.15)	0.450	
PR+	3795	0.88 (0.69-1.11)	1	1.11 (0.95-1.30)	1.47 (1.26-1.72)	1.88 (1.56-2.28)	< 0.001	0.260		¶		
PR-	1829	0.83 (0.61-1.14)	1	0.99 (0.73-1.34)	1.14 (0.87-1.49)	1.13 (0.86-1.48)	0.002	0.181	0.002‡	¶		
ER+PR+	3633	0.89 (0.68-1.16)	1	1.10 (0.93-1.29)	1.44 (1.23-1.69)	1.93 (1.55-2.40)	< 0.001	0.133		¶		
ER+PR-	852	0.85 (0.57-1.25)	1	1.13(0.84-1.53)	1.24(0.92-1.67)	1.17(0.86-1.61)	0.040	0.683		¶		
ER-PR-	906	0.90 (0.60-1.34)	1	0.97 (0.72-1.32)	1.20 (0.89-1.63)	1.25 (0.91-1.71)	0.011	0.869	0.014§	1.07 (1.01-1.13)	0.627	
**Postmenopausal, ever HRT users**												
Overall	9884	0.91 (0.82-1.01)	1	1.02 (0.94-1.10)	1.08 (0.99-1.17)	1.22 (1.12-1.33)	< 0.001	0.484		1.09 (1.06-1.12)	0.052	
ER+	6194	0.90 (0.78-1.03)	1	1.00 (0.90-1.10)	1.07 (0.96-1.18)	1.22 (1.09-1.36)	< 0.001	0.438		1.08 (1.05-1.11)	0.190	
ER-	1135	0.96 (0.68-1.34)	1	1.16 (0.89-1.50)	1.25 (0.96-1.62)	1.39 (1.06-1.83)	0.005	0.619	0.388†	1.12 (1.06-1.18)	0.323	0.255†
PR+	5064	0.89 (0.76-1.03)	1	1.03 (0.92-1.15)	1.08(0.96-1.21)	1.32 (1.17-1.50)	< 0.001	0.489		1.10 (1.07-1.14)	0.081	
PR-	1905	0.99 (0.78-1.26)	1	1.04 (0.83-1.31)	1.17 (0.97-1.41)	1.09(0.89-1.34)	0.116	0.856	0.114‡	1.04 (1.00-1.08)	0.546	0.021‡
ER+PR+	4912	0.89 (0.77-1.04)	1	1.02 (0.91-1.15)	1.08 (0.96-1.22)	1.32 (1.16-1.51)	< 0.001	0.383		1.10(1.06-1.14)	0.067	
ER+PR-	894	0.95 (0.69-1.31)	1	0.85 (0.66-1.10)	1.02 (0.79-1.32)	0.80 (0.60-1.07)	0.261	0.974		0.96 (0.91-1.02)	0.911	
ER-PR-	959	1.04(0.72-1.50)	1	1.21 (0.91-1.61)	1.32 (0.99-1.77)	1.46(1.08-1.97)	0.015	0.656	0.004§	1.10 (1.03-1.18)	0.140	<0.001§

* Adjusted for ethnicity (Caucasian, African-American, Hispanic, Asian, others), family history of breast cancer (yes, no), personal history of benign breast disease (yes, no), alcohol consumption (non-drinkers, >0-<5, 5-<15, 15-<30, ≥30 g/d), smoking status (never, past, current), education (<high school, high school, >high school), physical activity (low, medium, high, missing), age at menarche (<11, 11-12, 13-14, ≥15 yrs), height (<1.60, 1.60-<1.65, 1.65-<1.70, 1.70-<1.75, ≥1.75 m), oral contraceptive use (never, ever), energy intake (kcal/d, continuous), interaction between parity (0,1-2, ≥3) and age of first birth (<30, ≥ 30 yrs); age at baseline in years and year of questionnaire return were included as stratification variables.

† P-value for test for differences between ER- and ER+ subtypes.
‡ P-value for test for differences between PR- and PR+ subtypes.
§ P-value for test for differences between ER+PR+, ER+PR-, and ER-PR- subtypes (ER-PR+ had insufficient case numbers).
¶ Continuous estimates not shown because of significant nonlinearity in splines regression.
** P-value for test for interaction by menopausal status, categorical exposure: < 0.001.
†† P-value for test for interaction by HRT use, categorical exposure: < 0.001.

Table S6. Pooled multivariable-adjusted relative risks (RRs)* with (95% confidence intervals, CIs) of breast cancer for adult weight change between age 18-20 years and cohort baseline, stratified by BMI at age 18-20 years, in the Pooling Project of Prospective Studies of Diet and Cancer.

Breast cancer subtype per stratum of early adult BMI (18-20y)	Cases	Category of adult weight change (kg) - RRs (95%CIs)					*P* -value, test for trend	*P* -value, test for interaction between BMI at 18-20y and adult weight change
		< -2	-2 -< 2	2-<10	10-<20	≥20		
**Premenopausal**								
Total								
BMI18<21	1943	0.96 (0.73-1.27)	1	1.02 (0.88-1.18)	1.03 (0.88-1.21)	0.83 (0.68-1.02)	0.113	0.994
BMI18≥21	1189	0.75 (0.62-0.91)	0.84 (0.68-1.04)	0.85 (0.72-1.01)	0.86 (0.72-1.03)	0.68 (0.56-0.83)	0.256	
ER+								
BMI18<21	1104	0.95 (0.66-1.36)	1	1.00 (0.83-1.21)	0.91 (0.74-1.12)	0.77 (0.59-1.01)	0.053	0.363
BMI18≥21	682	0.73 (0.57-0.93)	0.84 (0.64-1.11)	0.78 (0.62-0.97)	0.93 (0.74-1.18)	0.57 (0.43-0.75)	0.057	
ER-								
BMI18<21	413	1.23 (0.68-2.20)	1	1.03 (0.74-1.44)	1.31 (0.92-1.86)	0.97 (0.62-1.50)	0.852	0.186
BMI18≥21	254	0.85 (0.56-1.28)	0.90 (0.56-1.45)	0.95 (0.65-1.38)	0.84 (0.56-1.28)	0.98 (0.64-1.50)	0.457	
**Postmenopausal, never HRT users**								
Total								
BMI18<21	5023	0.77 (0.59-1.01)	1	1.19 (1.02-1.37)	1.42 (1.23-1.65)	1.79 (1.55-2.07)	<0.001	0.018
BMI18≥21	3955	0.96 (0.81-1.13)	1.02 (0.85-1.23)	1.17 (1.01-1.36)	1.46 (1.26-1.70)	1.59 (1.37-1.85)	<0.001	
ER+								
BMI18<21	2781	0.65 (0.45-0.93)	1	1.09 (0.90-1.32)	1.39 (1.15-1.67)	1.77 (1.46-2.14)	<0.001	0.034
BMI18≥21	2204	0.92 (0.74-1.14)	0.98 (0.77-1.25)	1.14 (0.94-1.39)	1.44 (1.18-1.74)	1.57 (1.29-1.91)	<0.001	
ER-								
BMI18<21	658	1.01 (0.52-1.95)	1	1.19 (0.79-1.79)	1.38 (0.92-2.05)	1.66 (1.10-2.49)	<0.001	0.133
BMI18≥21	440	0.83 (0.52-1.34)	1.02 (0.62-1.69)	0.97 (0.64-1.48)	1.29 (0.85-1.96)	1.04 (0.68-1.61)	0.205	

* Adjusted for ethnicity (Caucasian, African-American, Hispanic, Asian, others), family history of breast cancer (yes, no), personal history of benign breast disease (yes, no), alcohol consumption (non-drinkers, >0-<5, 5-<15, 15-<30, ≥30 g/d), smoking status (never, past, current), education (<high school, high school, >high school), physical activity (low, medium, high, missing), age at menarche (<11, 11-12, 13-14, ≥15 yrs), height (<1.60, 1.60-<1.65, 1.65-<1.70, 1.70-<1.75, ≥1.75 m), oral contraceptive use (never, ever), energy intake (kcal/d, continuous), interaction between parity (0,1-2, ≥3) and age of first birth (<30, ≥ 30 yrs); age at baseline in years and year of questionnaire return were included as stratification variables.

Table S7. Funding and Acknowledgements for Individual Cohorts

Study Name (Acronym)	Funding Sources and Acknowledgements
Beta-Carotene and Retinol Efficacy Trial (CARET)	The CARET study was supported by U01 CA063673, R01 CA096789, UM1 CA167462, U01 CA167462.
Breast Cancer Detection Demonstration Project Follow-up Study (BCDDP)	The BCDDP was supported, in part, by the Intramural Research Program of the National Cancer Institute, National Institutes of Health.
California Teachers Study (CTS)	The CTS was supported by R01CA77398, U01CA199277 and UM1 CA169417.
Canadian National Breast Screening Study (CNBSS)	The CNBSS was supported by the Breast Cancer Research Foundation, BCRF-19-140.
Cancer Prevention Study-II Nutrition Cohort (CPS II)	The American Cancer Society funded the creation, maintenance and updating of the CPS-II cohort. We acknowledge the contribution to this study from central cancer registries supported through the Centers for Disease Control and Prevention's National Program of Cancer Registries, as well as cancer registries supported by the National Cancer Institute's Surveillance Epidemiology and End Results program. The views expressed here are those of the authors and do not necessarily represent the American Cancer Society or the American Cancer Society – Cancer Action Network.
CLUE II: Campaign Against Cancer and Heart Disease (CLUE II)	Funding for the CLUE II cohort was provided by grants from the National Cancer Institute (U01-CA-086308) and the National Institute on Aging (U01-AG-18033). Cancer incidence data were provided by the Maryland Cancer Registry, Center for Cancer Surveillance and Control, Maryland Department of Health, 201 W. Preston Street, Room 400, Baltimore, MD 21201. We acknowledge the State of Maryland, the Maryland Cigarette Restitution Fund, and the National Program of Cancer Registries of the Centers for Disease Control and Prevention for the funds that support the collection and availability of the cancer registry data.
Hormones and Diet in the Etiology of Breast Cancer Study (ORDET)	This study was supported by the Italian Association for Cancer Research (AIRC).
Iowa Women's Health Study (IWHS)	The IWHS study was funded by a grant from NCI grant R01 CA39742
Japan Public Health Center-Based Prospective Study Cohort 1 (JPHC I)	The JPHC study was supported by grants from the National Cancer Center Research and Development Fund (since 2011) and a Grant-in-Aid for Cancer Research from the Ministry of Health, Labour and Welfare of Japan (from 1989 to 2010).
Melbourne Collaborative Cohort Study (MCCS)	The MCCS cohort recruitment was funded by VicHealth and Cancer Council Victoria. The MCCS was further augmented by Australian National Health and Medical Research Council grants 209057, 396414 and 1074383 and by infrastructure provided by Cancer Council Victoria.
Multiethnic Cohort (MEC)	The MEC study was supported by a grant from the National Institutes of Health, National Cancer Institute: R37 CA54281 and U01 CA164973.
Netherlands Cohort Study (NLCS)	The NLCS was supported by grants from the Dutch Cancer Society and World Cancer Research Fund.
New York University Women's Health Study (NYUWHS)	This work was supported by grants from the National Institutes of Health (R01 CA098661, U01 CA182934 and center grants P30 CA016087 and P30 ES000260).
NIH-AARP Diet and Health Study (NIH-AARP)	AARP research was supported (in part) by the Intramural Research Program of the National Institutes of Health, National Cancer Institute.
Nurses' Health Study (NHS)	This work was supported by the National Institutes of Health grants UM1 CA186107, R01 CA49449 and P01 CA87969, the Breast Cancer Research Foundation. We would like to thank the following state cancer registries for their help: AL, AZ, AR, CA, CO, CT, DE, FL, GA, ID, IL, IN, IA, KY, LA, ME, MD, MA, MI, NE, NH, NJ, NY, NC, ND, OH, OK, OR, PA, RI, SC, TN, TX, VA, WA, WY. The authors assume full responsibility for analyses and interpretation of these data.

Study Name (Acronym)	Funding Sources and Acknowledgements
Nurses' Health Study II (NHS II)	This work was supported by the National Institutes of Health grants U01 CA176726, R01 CA50385 and the Breast Cancer Research Foundation. We would like to thank the following state cancer registries for their help: AL, AZ, AR, CA, CO, CT, DE, FL, GA, ID, IL, IN, IA, KY, LA, ME, MD, MA, MI, NE, NH, NJ, NY, NC, ND, OH, OK, OR, PA, RI, SC, TN, TX, VA, WA, WY. The authors assume full responsibility for analyses and interpretation of these data.
Prostate, Lung, Colorectal, and Ovarian Cancer Screening Trial USA (PLCO)	The PLCO study was supported by the Intramural Research Program of the Division of Cancer Epidemiology and Genetics and contracts from the Division of Cancer Prevention, National Cancer Institute, National Institutes of Health. The authors thank the PLCO screening center investigators and the staff from Information Management Services, Inc and Westat, Inc.
Swedish Mammography Cohort (SMC)	The SMC study was supported by grants from the Swedish Research Council and the Swedish Cancer Foundation.
Women's Health Study (WHS)	The WHS was supported by grants from the National Institutes of Health (CA047988, HL043851, HL080467, HL099355, CA182913).
Women's Lifestyle and Health Study (WLHS)	The WLHS was funded by Swedish Research Council (Vetenskapsrådet) grant K2012-69X-22062-01-3 and by the Swedish Cancer Society
